# Pathophysiology of fluid administration in critically ill patients

**DOI:** 10.1186/s40635-022-00473-4

**Published:** 2022-11-04

**Authors:** Antonio Messina, Jan Bakker, Michelle Chew, Daniel De Backer, Olfa Hamzaoui, Glenn Hernandez, Sheila Nainan Myatra, Xavier Monnet, Marlies Ostermann, Michael Pinsky, Jean-Louis Teboul, Maurizio Cecconi

**Affiliations:** 1 IRCCS Humanitas Research Hospital, Via Alessandro Manzoni 56, Rozzano, 20089 Milan, Italy; 2 Department of Biomedical Sciences, Humanitas University, Pieve Emanuele, Milan, Italy; 3 NYU Langone Health and Columbia University Irving Medical Center, New York, USA; 4 Erasmus MC University Medical Center, Rotterdam, The Netherlands; 5 Department of Anaesthesia and Intensive Care, Biomedical and Clinical Sciences, Linköping University, Linköping, Sweden; 6 Department of Intensive Care, CHIREC Hospitals, Université Libre de Bruxelles, Brussels, Belgium; 7 Service de Reanimation PolyvalenteHopital Antoine Béclère, Hopitaux Universitaires Paris-Saclay, Clamart, France; 8 Departamento de Medicina Intensiva, Facultad de Medicina, Pontificia Universidad Católica de Chile, Santiago, Chile; 9 Department of Anaesthesiology, Critical Care and Pain, Tata Memorial Hospital, Homi Bhabha National Institute, Mumbai, India; 10 Hôpitaux Universitaires Paris-Sud, Hôpital de Bicêtre, Medical Intensive Care Unit, Le Kremlin-Bicêtre, Paris, France; 11 Department of Intensive Care, King’s College London, Guy’s & St Thomas’ Hospital, London, UK; 12 Department of Critical Care Medicine, University of Pittsburgh, Pittsburgh, PA USA

## Abstract

Fluid administration is a cornerstone of treatment of critically ill patients. The aim of this review is to reappraise the pathophysiology of fluid therapy, considering the mechanisms related to the interplay of flow and pressure variables, the systemic response to the shock syndrome, the effects of different types of fluids administered and the concept of preload dependency responsiveness. In this context, the relationship between preload, stroke volume (SV) and fluid administration is that the volume infused has to be large enough to increase the driving pressure for venous return, and that the resulting increase in end-diastolic volume produces an increase in SV only if both ventricles are operating on the steep part of the curve. As a consequence, fluids should be given as drugs and, accordingly, the dose and the rate of administration impact on the final outcome. Titrating fluid therapy in terms of overall volume infused but also considering the type of fluid used is a key component of fluid resuscitation. A single, reliable, and feasible physiological or biochemical parameter to define the balance between the changes in SV and oxygen delivery (i.e., coupling “macro” and “micro” circulation) is still not available, making the diagnosis of acute circulatory dysfunction primarily clinical.

## Take-home messages

- Fluids are drugs used in patients with shock to increase the cardiac output with the aim to improve oxygen delivery to the cells. The response to fluid administration is determined by the physiological interaction of cardiac function and venous return. In septic shock, the beneficial clinical response of fluid administration is rapidly reduced after few hours and fluid titration is crucial to avoid detrimental fluid overload. The fluid challenge is a fluid bolus given at a defined quantity and rate to assess fluid responsiveness.
- The ideal fluid for critically ill patients does not exist; however, crystalloids should be used as first choice. Balanced crystalloid solutions may be associated with better outcomes but the evidence is still low. Albumin infusion may have a role in already fluid resuscitated patients at risk of fluid overload.
- Fluid administration is integrated into the complex management of pressure and flow “macro” hemodynamic variables, coupled to the “micro” local tissue flow distribution and regional metabolism. Macro-variables are managed by measuring systemic blood pressure and evaluating the global cardiac function. The critical threshold of oxygen delivery to the cells is difficult to estimate, however, several indexes and clinical signs may be considered as surrogate of that, and integrated in a decision-making process at the bedside.

## Background

Fluid administration is one of the most common but also one of the most disputed interventions in the treatment of critically ill patients. Even more debated is the way how to appraise and manage the response (in terms of flow and pressure variables) to fluid administration, which ranges from a prosaic “just give fluids” to the fluid challenge, to the evaluation of fluid responsiveness before fluid administration to, finally, recent approaches based on machine learning and Artificial Intelligence aimed at personalizing its use [1–3].

Shock occurs in many intensive care unit (ICU) patients, representing a life-threatening condition that needs both prompt recognition and treatment to provide adequate tissue perfusion and thus oxygen delivery to the cells [4]. A large trial in more than 1600 patients admitted to ICU with shock and requiring vasopressors demonstrated that septic shock was the most frequent type of shock, occurring in 62% of patients, while cardiogenic shock (16%), hypovolemic shock (16%) and other types of distributive (4%) or obstructive (2%) shock were less frequent. The progression of this syndrome is associated with mitochondrial dysfunction and deregulated cell-signaling pathways, which can lead to multiple organ damage and failure and, eventually, untreatable hemodynamic instability and death [5].

Optimal treatment of shock is time-dependent and requires prompt and adequate combined support with fluids and/or vasopressors [4, 6–8]. The rationale, supported by robust evidence from several physiological and clinical studies, is to improve oxygen delivery (DO_2_), so that systemic oxygen requirements can be met [4, 6]. Oxygen delivery is defined as the product of oxygen content and cardiac output (CO). Pathological cellular oxygen utilization results from a tissue oxygen request exceeding the DO_2_ or the cellular inability to use O_2_. Our understanding of the mechanisms of shock has improved in the last decades, shifting the clinical practice from a “one size fits all” policy to individualized management [4, 9, 10].

Fluids are the first line of treatment in critically ill patients with acute circulatory failure aiming to increase venous return, stroke volume (SV) and, consequently, CO and DO_2_ [4]. The effect of the increase in CO following fluid resuscitation on blood pressure is not linear and related to baseline conditions (see "Fluids and ICU outcomes: does the type of fluid matter?") [4, 11–15].

Dr Thomas Latta first described the technique of fluid resuscitation to treat an episode of shock in 1832 in a letter to the editor of *The Lancet* [16]. He injected repeated small boluses of a crystalloid solution to an elderly woman and observed that the first bolus did not produce a clinically relevant effect; however, after multiple boluses (overall 2.8 L) ‘soon the sharpened features, sunken eye, and fallen jaw, pale and cold, bearing the manifest imprint of death's signet, began to glow with returning animation; the pulse returned to the wrist’. This lady was ultimately the first fluid responder reported in the literature.

This meaningful witness from the past addresses several physiological and clinical issues, which are still valid after almost 200 years:

CO is the dependent variable of the physiological interaction of cardiac function (described by the observations of Otto Frank and Ernest Starling more than 100 years ago) [17] and venous return (based on Guyton’s relationship between the elastic recoil of venous capacitance vessels, the volume stretching the veins, the compliance of the veins and the resistance of the venous system) [18, 19]. Fluid responsiveness indicates that the heart of the patient is operating in the steep part of Frank–Starling’s curve of heart function, while fluid non-responsiveness is observed on the flat part of the curve where an increase in preload doesn't increase CO further [20–22]. The lady treated by Dr. Latta probably did not respond to the first fluid bolus because the volume infused was insufficient to increase venous return (i.e., induce a change in the stressed volume related to venous compliance) [18, 19]. Thus, the volume infused is a crucial factor. The most recent Surviving Sepsis Campaign guidelines again recommend to administer an initial fluid volume of at least 30 ml/kg in patients with sepsis, which is considered, on average, a safe and effective target [6]. However, as the goal of fluid therapy is to increase SV and then CO, fluids should only be given if the plateau of cardiac function has not been reached in the individual patient. In fact, and probably even before reaching this point, fluid administration that does not increase CO can be considered futile. The fluid challenge (FC) is a hemodynamic diagnostic test consisting of the administration of a fixed volume of fluids with the purpose of identifying fluid responsive patients who will increase CO in response to fluid infusion [12, 23, 24]. This approach allows the individual titration of fluids and reduces the risk of fluid overload, which affects patients’ clinical outcome and mortality [9, 25–27].

In clinical practice, the likelihood of a beneficial clinical response to an FC is rapidly reduced after a few hours following the onset of septic shock resuscitation which renders the optimization of fluid therapy quite complex without adopting hemodynamic monitoring and resuscitation targets (i.e., CO increase above predefined thresholds) [28].

Fluid administration in responsive shock patients is associated with clinically evident signs of restored organ perfusion. Hence, administering fluids during shock and observing the patient’s clinical improvement at the bedside has proven to be reasonable since 1832. Does the target matter? There is no single clinical or laboratory variable that unequivocally represents tissue perfusion status. Therefore, a multimodal assessment is recommended [4]. Several aspects should be taken into account when identifying a variable as a potential trigger or target for fluid resuscitation, but most importantly the variable has to be flow-sensitive [29]. This means choosing a variable that exhibits an almost real-time response to increases in systemic blood flow and/or perfusion pressure and may be suitable to assess the effect of a fast-acting therapy such as a fluid bolus over a very short period of time (e.g., 15 min) [30]. Persistent hyperlactatemia may not be an adequate trigger since it has multiple aetiologies, including some that are non-perfusion related in many patients [e.g., hyperadrenergism or liver dysfunction], and pursuing lactate normalization may thus increase the risk of fluid overload [31]. Indeed, a recent study showed that systemic lactate levels remained elevated in 50% of a cohort of ultimately surviving septic shock patients. In contrast, flow-sensitive variables such as peripheral perfusion, central venous O_2_ saturation, and venous–arterial pCO_2_ gradients were normal in almost 80% of patients at two hours [32]. Peripheral perfusion, as represented by capillary refill time (CRT), appears to be a physiologically sound variable to be used as a trigger and a target for fluid resuscitation. A robust body of evidence confirms that abnormal peripheral perfusion after early [33] or late [34–36] resuscitation is associated with increased morbidity and mortality. A cold, clammy skin, mottling and prolonged CRT have been suggested as triggers for fluid resuscitation in patients with septic shock. Moreover, the excellent prognosis associated with a normal CRT or its recovery, its rapid response time to fluid loading, relative simplicity, availability in resource-limited settings, and its capacity to change in parallel with perfusion of physiologically relevant territories such as the hepatosplanchnic region [37], are strong reasons to consider CRT as a target for fluid resuscitation in septic shock patients. A recent major randomized controlled trial (RCT) demonstrated that CRT-targeted resuscitation was associated with lower mortality, less organ dysfunction, and lower treatment intensity than a lactate-targeted one, including less resuscitation fluids [38, 39]. Septic shock is characterized by a combination of a decrease in vascular tone, affecting both arterioles and venules, myocardial depression, alteration in regional blood flow distribution and microvascular perfusion and increased vascular permeability. Moreover, macro- and micro-circulation are physiologically regulated to maintain the mean arterial pressure (MAP) by adapting the CO to the local tissue flow distribution, which is associated with regional metabolism. From a clinical perspective, once normal organ perfusion is achieved, the rationale for augmenting macro hemodynamic variables (MAP and CO) by giving fluids or vasopressors, is quite low.

The lady's pulse “returned to the wrist”, implying that flow and pressure responses in that patient were linked. In daily practice, hypotension is frequently used to trigger fluid administration. The MAP target is also used by many ICU physicians as an indicator to stop fluid infusion [40]. This assumption is flawed in many aspects. First, restoring MAP above predetermined targets does not necessarily mean reversing shock; similarly, a MAP value below predefined thresholds does not necessarily indicate shock [4]. Second, and more importantly, the physiological relationship between changes in SV and changes in MAP is not straightforward and depends on vascular tone and arterial elastance. In patients with high vasomotor tone, an increase in SV after fluid administration will be associated with an increase in MAP. This is typically the case in patients with pure hypovolemia, such as hemorrhagic shock, in whom the physiologic response to hemorrhage includes severe venous and arterial vasoconstriction. In patients with low vasomotor tone, such as in sepsis but also during deep anesthesia, MAP hardly changes after fluid administration even though SV may markedly increase. The lack of a significant relationship between MAP and SV has been demonstrated in many ICU patients, especially during septic shock [41–43]. Interestingly, dynamic arterial elastance (computed as respiratory changes in pulse pressure divided by changes in SV) can be used to identify patients who are likely to increase their MAP in response to fluids [44, 45], but this requires specific monitoring tools. Finally, one should recall that the main purpose of fluid administration is to increase tissue perfusion, and hence changes in MAP should be considered as beneficial but not regarded as the main target for fluid administration [46].

Since human physiology has remained consistent over the centuries, the mechanisms in the interplay of flow and pressure, the systemic response of these variables to the shock syndrome, the effects of fluid administration and the concept of preload dependency and preload responsiveness are still valid. This paper aims to integrate these physiological concepts with recent advances related to three main pathophysiological aspects of fluid administration in ICU patients.

### The fluid challenge, fluid bolus and fluid infusion: does the rate of administration matter?

According to the Frank–Starling law, there is a curvilinear relationship between preload (the end-diastolic transmural pressure) and the generated SV, which is affected by the inotropic condition of the heart muscle (for a given preload, increased inotropy would enhance the response and, hence, the SV, and vice versa). The curve is classically subdivided into two zones that can be distinguished: (1) a steep part where small preload changes produce a marked increase in SV (preload dependent zone) and (2) a flat part where the SV is minimally or not affected by preload changes (preload independence zone).

The physiological link behind the described relationship between preload, SV and fluid administration is that the volume infused has to be large enough to increase the driving pressure for venous return, and that the resulting increase in end-diastolic volume produces an increase in SV only if both ventricles are operating on the steep part of the curve. Accordingly, the FC may be defined as the smallest volume required to efficiently challenge the system. Thus, the only reason to give fluids during resuscitation of circulatory shock is to increase the mean systemic pressure with the aim to increase the driving pressure for venous return (defined as mean systemic pressure minus right atrial pressure), as shown in a recent prospective study exploring the cardiovascular determinants of the response to resuscitation efforts in septic patients [47]. Most FC will increase mean systemic pressure, if given in large enough volumes and at a fast enough rate as described below. However, a simultaneous increase in right atrial pressure suggests that the subject is not volume responsive, and their preload responsiveness status needs to be reassessed.

Considering the FC as a drug (e.g., study the response by applying a pharmacodynamic methodology) has been the topic of only a few studies. The first small-sized study conducted by Aya et al. in postoperative patients, demonstrated that the minimum volume required to perform an effective FC was 4 ml/kg [48]. However, in the literature, most of the studies in the field of fluid responsiveness and FC response in ICU patients adopt a volume of 500 ml (on average) [49], which is largely above 4 ml/kg for the vast majority of ICU patients. Interestingly, 500 mL was also the median volume administered in clinical practice in the FENICE study (an observational study including 311 centers across 46 countries) [40], whereas a lower mean volume (250 ml) is usually used in high-risk surgical patients undergoing goal-directed therapy optimization [50]. This difference may imply that a larger fluid bolus is often adopted not just to assess fluid responsiveness but also to treat an episode of hemodynamic instability, implying a therapeutic effect of fluid administration. Since, the use of repetitive fluid boluses may increase the risk of fluid overload, the prediction of fluid responsiveness prior to FC administration is a key point which, unfortunately, remains challenging [4, 51–54]. In fact, several bedside clinical signs, systemic pressures and static volumetric variables adopted in the clinical practice at the bedside are poorly predictive of the effect of FC infusion [53–55]. To overcome these limitations, bedside functional hemodynamic assessment has gained in popularity, consisting of a maneuver that affects cardiac function and/or heart–lung interactions, with a subsequent hemodynamic response, the extent of which varies between fluid responders and non-responders [53–56].

Recently, all aspects related to FC administration were investigated, showing that the amount of fluid given, the rate of administration and the threshold adopted to define fluid responsiveness impact on the final outcome of an FC [57–61]. A RCT showed that the duration of the administration of an FC affected the rate of fluid responsiveness, shifting from 51.0% after a 4 ml/kg FC completed in 10 min to 28.5% after an FC completed in 20 min [57]. However, this study was conducted in a limited sample of neurosurgical patients during a period of hemodynamic stability, which limits the external validity of the results in different surgical settings or in critically ill patients.

What would be the best rate of infusion when boluses of fluid are given without using the FC technique? It has been postulated that slower rates may limit vascular leakage due to a less abrupt increase in hydrostatic pressure. Recently, a large multicentric trial randomized 10,520 patients to receive fluids at an infusion rate reflecting current standard of care [a fluid bolus of 500 ml over approximately 30 min, i.e*.,* the upper limit of infusion rate for infusion pumps (999 ml/h; 16 ml/min)] versus a slower infusion rate (333 ml/h; 5.5 ml/min), which reflects less than the 25% percentile in FENICE cohort study [62]. Importantly, the rates adopted in this trial were overall slower than those adopted in clinical studies where the FC is used to correct hemodynamic instability (i.e*.,* 500 ml in 10 min = 50 ml/min; 500 ml in 20 min = 25 ml/min), suggesting that the authors applied a fluid bolus, just not at the “correct” rate. Neither the primary outcome (90-day mortality), nor all of the secondary clinical outcomes during the ICU stay were different between the two groups, suggesting that the infusion rate of continuous fluid administration for fluid expansion does not affect clinical outcomes [62]. This was not unexpected as only the administration rate differed, while the total amount of fluids was identical and the proportion of volume responsive patients was probably also similar in the two groups (even though not measured, this proportion is assumed to be identical as per the effects of randomization in large groups).

### Fluids and ICU outcomes: does the type of fluid matter?

The ideal fluid for patients in shock should have a composition similar to plasma to support cellular metabolism and avoid organ dysfunction and should be able to achieve a sustained increase in intravascular volume to optimize CO. Unfortunately, no ideal fluid exists. The available fluid options are broadly divided into three groups: crystalloids, colloids, and blood products. The latter have few very specific indications, including shock in trauma patients and hemorrhagic shock, and will not be discussed in this review.

Colloids are composed of large molecules designed to remain in the intravascular space for several hours, increasing plasma osmotic pressure and reducing the need for further fluids. Despite their theoretical advantages, patients with sepsis often have alterations in glycocalyx and increased endothelial permeability, which may lead to extravasation of colloids’ large molecules [63, 64], increases the risk of global increased permeability syndrome and abolishes the primary advantage [65]. Colloids are further divided into semisynthetic colloids and albumin. Semisynthetic colloids include hydroxyethyl starches, dextrans and gelatins, which have demonstrated either no effect [66] or detrimental consequences in critically ill patients, increasing the risk of acute kidney injury (AKI) [67, 68]. Thus, the use of semisynthetic colloids in shock patients should be abandoned.

Albumin is distributed in intravascular and extravascular fluid. In health, up to 5% of intravascular albumin leaks per hour into the extravascular space [transcapillary escape rate (TER)] giving a distribution half-time of about 15 h. This rate may increase up to 20% or more in septic shock. Accordingly, the measured TER of albumin to the tissues (the so-called “TCERA”) is said to be an index of ‘vascular permeability [69].

The role of albumin for fluid therapy is still debated (reference 64). Although theoretically promising for its anti-inflammatory and anti-oxidant proprieties [70], and for its supposedly longer intravascular confinement due to the interaction between its surface negative charges and the endovascular glycocalyx [70], clinical data have been conflicting [30, 71]. While the use of albumin was associated with improved MAP, the relative risk of mortality was similar to crystalloid infusion [71]. A predefined subgroup analysis of the ‘Comparison of Albumin and Saline for Fluid Resuscitation in the Intensive Care Unit’ (SAFE) study suggested that albumin should be avoided in patients with traumatic brain injury. In contrast, albumin is recommended for patients with chronic liver disease and in combination with terlipressin for patients with hepatorenal syndrome [72, 73]. The most recent Surviving Sepsis Guidelines also suggest using albumin in patients with sepsis who have received large volume crystalloid resuscitation [6].

On the other waterside of fluid therapy, crystalloids are composed of water and electrolytes [74]. Saline 0.9% was the first crystalloid solution to be utilized in humans. Its drawbacks are an unphysiological concentration of chloride and sodium and high osmolarity, which have been associated with nephrotoxicity and hyperchloremic acidosis [75]. Extracellular chloride influences the tone of the afferent glomerular arterioles, directly impacting the glomerular filtration rate (GFR). Several balanced solutions have since been introduced, such as Ringer’s lactate (Hartmann’s solution), Ringer’s acetate and Plasmalyte. These solutions have a lower chloride concentration and lower osmolarity (between 280 and 294mosm/l) and are buffered with lactate or acetate to maintain electroneutrality. In healthy adult human volunteers, infusion of 2 l of saline 0.9% versus a balanced crystalloid solution decreased urinary excretion of water and sodium [76].

Several recent RCTs assessed the effect of balanced solutions vs saline 0.9% in critically ill patients (Table 1). The SPLIT trial, conducted in 4 ICUs, showed no advantage in either group [77]. The SMART trial was a monocentric study (5 ICUs in 1 academic center) comparing Plasmalyte versus saline 0.9% in critically ill patients admitted to ICU [78]. A significant difference in favor of Plasmalyte was found in the composite outcome MAKE30 consisting of death from any cause, new renal replacement therapy or persistent renal dysfunction within 30 days [78]. The Plasma-Lyte 148^®^ versus Saline (PLUS) study was a blinded RCT in 5037 adult patients expected to stay in the ICU for at least 72 h and needing fluid resuscitation [79]. Patients with traumatic brain injury or at risk of cerebral edema were excluded. There was no significant difference in 90-day mortality or AKI between both groups. Similarly, the ‘Balanced Solutions in Intensive Care Study’ (BaSICS), a multi-center double-blind RCT comparing the same fluid solutions in 11,052 patients in 75 ICUs across Brazil, found no significant difference in mortality or renal outcomes [62]. An updated meta-analysis of 13 high-quality RCTs, including the PLUS and BaSICS trials, concluded that the balanced crystalloid effect ranged from a 9% relative reduction to a 1% relative increase in mortality with a similar decrease in risk of AKI [80].

**Table 1:** Recent randomized controlled trials comparing saline 0.9% versus balanced crystalloids

Study	SPLIT [77]	SMART [78]	BaSICS [62]	PLUS [79]
Setting	4 ICUs in New Zealand	5 ICUs in single center in USA	75 ICUs in Brazil	53 ICUs in Australia and New Zealand
Study design	Double-blind, cluster-randomized, double-crossover trial	Open-label, cluster-crossover trial	Double-blind, factorial, randomized clinical trial	Double-blind randomized controlled trial
Number of participants	2,278	15,802	11,052	5,037
Population	Critically ill adults (mainly surgical)	Critically ill adults	Critically ill adults (~ 50% elective surgery)	Critically ill adult patients (expected to stay in the ICU for at least 72 h)
Intervention	Plasmalyte	RLS/Plasmalyte	Plasmalyte	Balanced multielectrolyte solution
Control	0.9% NaCl	0.9% NaCl	0.9% NaCl	0.9% NaCl
Primary outcome (intervention vs control)	AKI (9.6% vs 9.2%; *p* = 0.77)	MAKE30 (14.3% vs 15.4%; *p* = 0.04)	90-day mortality (26.4% vs 27.2%; *p* = 0.47)	90-day mortality (21.8% vs 22%; *p* = 0.90)
Secondary outcomes (intervention vs control)	In-hospital mortality (7.6% vs 8.6%) RRT (3.3% vs 3.4%)	In-hospital mortality (25.2% vs 29.4%) RRT (2.5% vs 2.9%)	AKI with RRT (0.88% vs 0.93%) NeuroSOFA > 2 (32.1% vs 26%)	New RRT (12.7% vs 12.9%) No significant difference in maximum increase in serum creatinine

*ICU* intensive care unit, *RLS* ringer-lactate solution, *AKI* acute kidney injury, MAKE30 clinical outcome consisting of death from any cause, new renal replacement therapy or persistent renal dysfunction within 30 days, *NaCl* saline solution, *RRT* renal replace therapy, *SOFA* sequential organ failure assessment score

A possible cofounding factor of trials investigating the effect of different types of crystalloids on the final outcome could be related to the volume and type of fluid administration prior to enrollment. In fact, a secondary post hoc analysis of the BaSICS trial categorized the enrolled patients according to fluid use in the 24 h before enrollment and according to admission type, showing a high probability that 90-day mortality was reduced in patients who exclusively received balanced fluids [81].

Overall, considering that balanced solutions in sepsis may be associated with improved outcomes compared with chloride-rich solutions and the lack of cost-effectiveness studies comparing balanced and chloride-rich crystalloid solutions**,** balanced crystalloids are recommended (weak recommendation) as first-line fluid type in patients with septic shock [6, 78].

### Fluid administration response during acute circulatory failure

Prompt fluid resuscitation in the early phase of acute circulatory failure is a key recommended intervention [11, 82]. On the other hand, the hemodynamic targets and the safety limits indicating whether to stop this treatment in already resuscitated patients are relatively undefined and poorly titratable to the specific patient response [9, 82]. However, targeted fluid management is of pivotal importance to improve the outcome of hemodynamically unstable ICU patients since both hypovolemia and hypervolemia are harmful [4]. Acute circulatory dysfunction is often approached by using a fluid resuscitation, with the purpose of optimizing the CO to improve the DO_2_. However, a single, reliable, and feasible physiological or biochemical parameter to define the balance between the changes in CO and in DO_2_ (i.e., coupling “macro” and “micro” circulation) is still not available, making the diagnosis of acute circulatory dysfunction primarily clinical [3].

However, recognizing the value of the CO itself or tracking its changes after fluid administration, is poorly associated with the variables usually evaluated at the bedside. In fact, the ability of ICU physicians to estimate the exact CO value based on clinical examination is rather low (i.e., 42–62% of the cases), often leading to incongruent evaluations (meaning that the CO was estimated as increased, whereas the real CO was decreased, or vice versa) [83].

The role of echocardiography in ICU has changed in the last decades with more focus on the characteristics of the individual patient. “Critical care echocardiography” (CCE) is performed and interpreted by the intensivists 24/7 at the bedside, to help diagnosing the type of shock, to guide therapy according to the type of shock and, finally, to customize the therapy at the bedside by re-evaluating the strategies adopted [84, 85]. The global adoption of CCE has been hampered by technical problems (portability and availability of the machines), and by a lack of formal training programs for CCE. These gaps have been recently filled by technical progress providing high-quality images at the bedside, and by new guidelines for skills certification, developed by the European Society of Intensive Care Medicine [86], the American [87] and the Canadian Society of Echocardiography [88], and training standards [89]. CCE should now be considered as a part of the routine assessment of ICU patients with hemodynamic instability since assessment of cardiac function plays a central role in therapy.

The CCE-enhanced clinical evaluation of hemodynamically unstable patients should be coupled with clinical variables evaluating the relationship between DO_2_ and oxygen consumption. In fact, although the exact value of “critical” DO_2_ is difficult to estimate, the systemic effects of overcoming this threshold can be recognized.

The CRT measures the time required to recolor the tip of a finger after pressure is applied to cause blanching. Since this maneuver depends on the applied pressure, Ait-Oufella et al. recommended to use just enough pressure to remove the blood at the tip of the physician’s nail illustrated by appearance of a thin white distal crescent under the nail, for 15 s [36]. CRT at 6 h after initial resuscitation was strongly predictive of 14-day mortality (area under the curve of 84% [IQR: 75–94]). Hernandez et al. reported that CRT < 4 s, 6 h after resuscitation was associated with resuscitation success, with normalization of lactate levels 24 h after the occurrence of severe sepsis/septic shock [90]. A prospective cohort study of 1320 adult patients with hypotension in the emergency room, showed an association between CRT and in-hospital mortality [91].

Serum lactate is a more objective metabolic surrogate to guide fluid resuscitation. Irrespectively of the source, increased lactate levels are associated with worse outcomes [92], and lactate-guided resuscitation significantly reduced mortality as compared to resuscitation without lactate monitoring [93]. Since serum lactate is not a direct measure of tissue perfusion [94], a single value is less informative than the trend of lactate clearance. However, serum lactate normalization is indicative of shock reversal whereas severe hyperlactatemia is associated with very poor outcomes. Recent published data showed that lactate levels > 4 mmol/l combined with hypotension are associated with a mortality rate of 44.5% in ICU patients with severe sepsis or septic shock [92]. For instance, a large retrospective study showed that a subgroup of ICU patients with severe hyperlactatemia (lactate > 10 mmol/l) had a 78.2% mortality, which increased up to 95% if hyperlactatemia persisted for more than 24 h [95].

ScvO_2_ reflects the balance between oxygen delivery and consumption, being a surrogate value of mixed venous oxygen saturation (normally the ScvO_2_ is 2–3% lower than SvO_2_) [96]. It was previously considered as a therapeutic target in the management of early phases of septic shock [14, 97, 98] but this approach has been challenged by the negative results of three subsequent large multicentric RCTs [99–101] and is no longer recommended [82]. However, since the ARISE, PROMISE and the PROCESS trials probably included populations of less severe critically ill patients compared to the study by Rivers et al. [97] (i.e*.,* lower baseline lactate levels, ScvO_2_ at or above the target value at the admission, and lower mortality in the control group) [99–101], the normalization of low ScvO_2_ in the early phase of septic shock can be still considered a good goal of successful resuscitation. While the incidence of low ScvO_2_ in current practice is low [102], the persistence of high values of ScvO_2_ is associated with mortality in septic shock patients, probably indicating an irreversible impairment of oxygen extraction by the cells [69].

The venous-to-arterial CO_2_ tension difference (ΔPCO_2_) and central venous oxygen saturation (ScVO_2_) provide adjunctive relevant clinical information. It is obtained by measuring central venous PCO_2_ sampled from a central vein catheter and arterial PCO_2_ and strongly correlates with the venous-to-arterial CO_2_ tension difference [P (v-a) CO_2_], which is the gradient between PCO_2_ in mixed venous blood (PvCO_2_ measured with pulmonary artery catheter) and PCO_2_ in arterial blood (PaCO_2_): P(v-a)CO_2_ = PvCO_2_-PaCO_2_ [103]. This point is crucial because the results of several studies in the past analyzing the changes of P(v-a) CO_2_ during shock emphasize that it is still useful to measure central venous PCO_2_ instead of mixed venous blood. In health, ΔPCO_2_ ranges between 2 and 6 mmHg.

The pathophysiological background of the determinants of this index is rather complex but ΔPCO_2_ changes in a shock state are coupled with other indices of tissue perfusion. First of all, according to a modified Fick equation, ΔPCO_2_ is linearly linked to CO_2_ generation and inversely related to CO [104]. Several clinical studies confirmed both the strong association between CO and P (v-a) CO_2_ and between impairment in microcirculatory perfusion and tissue PCO_2_ [105]. Accordingly, an elevated ΔPCO_2_ may be due to either a low CO state or to an insufficient microcirculation to remove the additional CO_2_ in hypoperfused tissues despite an adequate CO.

These conditions may be further investigated by coupling the information obtained by ΔPCO_2_ and ScVO_2_. In fact, an increased ΔPCO_2_ associated with a decreased ScVO_2_ is suggestive of a low CO, whereas a normal/high ScVO_2_ associated with an increased ΔPCO_2_ indicates impaired tissue perfusion. Pragmatically, a normal ∆PCO_2_ (< 6 mmHg) value in a shocked patient should defer from increasing the CO as first step; instead, regional blood flow may be impaired even in presence of a normal/high CO.

**Fig. 1:**
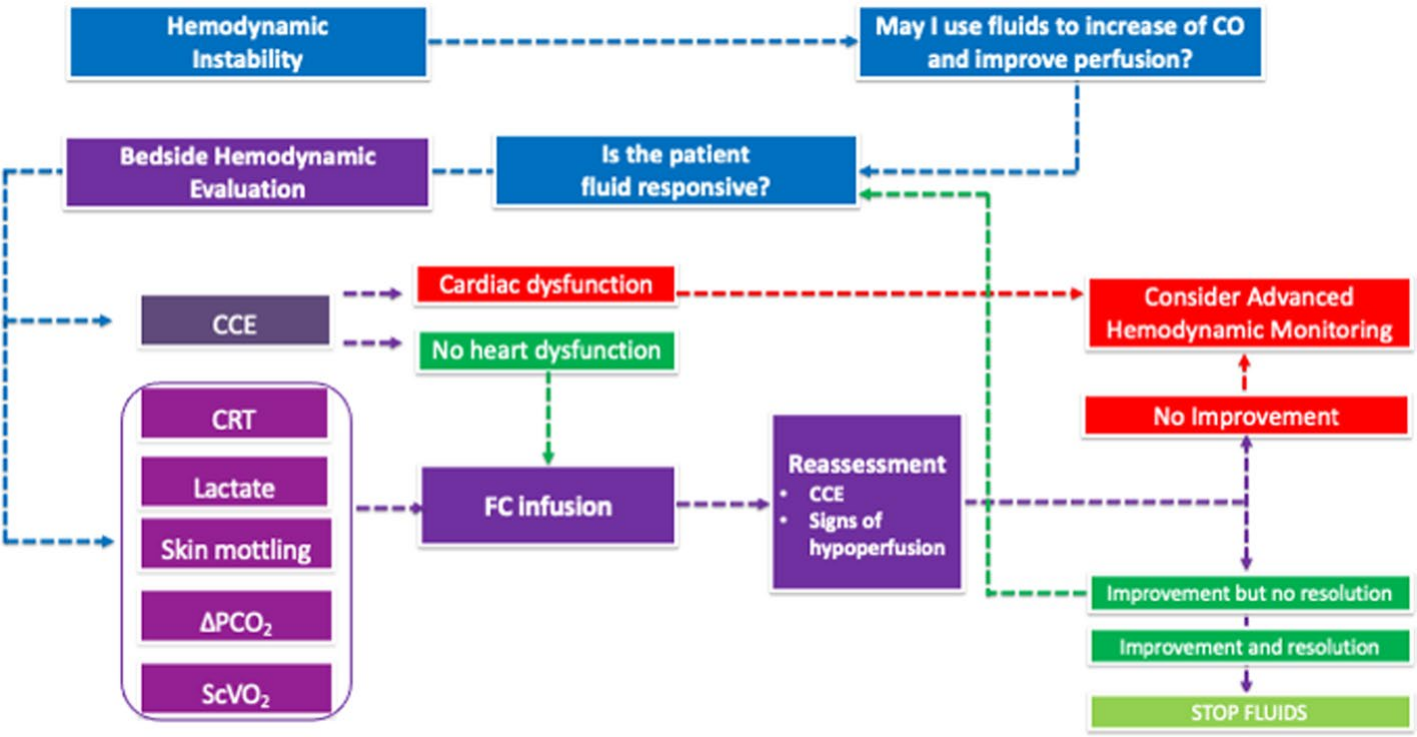
Decision-making process at the bedside to guide and titrate fluid administration during an episode of acute circulatory failure. *CCE* critical care echocardiography, *CO* cardiac output, *CRT* capillary refill time, *FC* fluid challenge, *ΔPCO*_*2*_ venous-to-arterial CO_2_ tension difference, *ScVO*_*2*_ central venous oxygen saturation

All these aspects may be integrated into a decision-making algorithm where the clinical signs of hypoperfusion are coupled with the CCE evaluation (Fig. 1). The clinical recognition of signs of systemic hypoperfusion should trigger the use of fluids with the purpose of optimizing the CO and improving the DO_2_. This choice recognizes fluids as drugs that should only be used as long as the effect on CO is likely. Monitoring fluid responsiveness during the resuscitation phase of an episode of acute circulatory failure may be achieved by applying a “closed-loop” operative strategy, where the signs of tissue hypoperfusion and the findings of CCE are re-evaluated after each fluid bolus. More sophisticated tools are useful when the cardiovascular system reaches the plateau of clinical response or, earlier, when the CCE shows acute or acute-on-chronic cardiac dysfunction at the baseline examination of the patient.

## Conclusions

The physiology of fluid administration in critically ill patients is of major importance in ICU. With a solid basis in the dynamic and complex balance between cardiovascular function and systemic response, fluids should be considered as drugs and intensivists should consider their pharmacodynamic and biochemical properties to optimize the therapy. A multimodal approach is required since single physiological or biochemical measurements able to adequately assess the balance between the CO and tissue perfusion pressure are still lacking. The assessment of response to fluid administration may be obtained by coupling the changes of different signs of tissue hypoperfusion using clinical and invasive hemodynamic monitoring, with the evaluation of cardiac function based on critical care echocardiography.

## Data Availability

Not applicable.
